# Simian immunodeficiency virus-infected rhesus macaques with AIDS co-develop cardiovascular pathology and encephalitis

**DOI:** 10.3389/fimmu.2023.1240946

**Published:** 2023-10-27

**Authors:** Kevin S. White, Joshua A. Walker, John Wang, Patrick Autissier, Andrew D. Miller, Nadia N. Abuelezan, Rachel Burrack, Qingsheng Li, Woong-Ki Kim, Kenneth C. Williams

**Affiliations:** ^1^ Department of Biology, Boston College, Chestnut Hill, MA, United States; ^2^ Department of Biomedical Sciences, Section of Anatomic Physiology, Cornell University College of Veterinary Medicine, Ithaca, NY, United States; ^3^ Connel School of Nursing, Boston College, Chestnut Hill, MA, United States; ^4^ Nebraska Center for Virology, School of Biological Sciences, University of Nebraska-Lincoln, Lincoln, NE, United States; ^5^ Division of Microbiology, Tulane National Primate Research Center, Covington, LA, United States

**Keywords:** HIV, monocyte/macrophage, pathology, co-morbidities, AIDS

## Abstract

Despite effective antiretroviral therapy, HIV co-morbidities remain where central nervous system (CNS) neurocognitive disorders and cardiovascular disease (CVD)-pathology that are linked with myeloid activation are most prevalent. Comorbidities such as neurocogntive dysfunction and cardiovascular disease (CVD) remain prevalent among people living with HIV. We sought to investigate if cardiac pathology (inflammation, fibrosis, cardiomyocyte damage) and CNS pathology (encephalitis) develop together during simian immunodeficiency virus (SIV) infection and if their co-development is linked with monocyte/macrophage activation. We used a cohort of SIV-infected rhesus macaques with rapid AIDS and demonstrated that SIV encephalitis (SIVE) and CVD pathology occur together more frequently than SIVE or CVD pathology alone. Their co-development correlated more strongly with activated myeloid cells, increased numbers of CD14+CD16+ monocytes, plasma CD163 and interleukin-18 (IL-18) than did SIVE or CVD pathology alone, or no pathology. Animals with both SIVE and CVD pathology had greater numbers of cardiac macrophages and increased collagen and monocyte/macrophage accumulation, which were better correlates of CVD-pathology than SIV-RNA. Animals with SIVE alone had higher levels of activated macrophage biomarkers and cardiac macrophage accumulation than SIVnoE animals. These observations were confirmed in HIV infected individuals with HIV encephalitis (HIVE) that had greater numbers of cardiac macrophages and fibrosis than HIV-infected controls without HIVE. These results underscore the notion that CNS and CVD pathologies frequently occur together in HIV and SIV infection, and demonstrate an unmet need for adjunctive therapies targeting macrophages.

## Introduction

HIV-associated comorbidities affect 20-50% of people living with HIV (PLWH) despite effective antiretroviral therapy (ART) (1–6). Of these, cardiovascular diseases (CVD) and HIV-associated neurocognitive disorders (HAND) are the most prevalent and are likely linked, although this has not been thoroughly documented (7–11). Traditional biomarkers of CVD are insufficient for predicting CVD risk in HIV-infected individuals on ART, highlighting the necessity to further investigate the etiologies of HIV-associated CVD pathogenesis (2, 5, 12, 13). Anecdotally, it appears that the incidence of cardiac and central nervous system (CNS) pathologies, and of CVD and neurocognitive dysfunction among HIV-uninfected individuals often are concomitant and are linked; likely through systemic inflammation and cardiovascular risk factors (14–21). Importantly, both are associated with increased monocyte/macrophage activation and accumulation in tissues (22–26). The development of pre-ART HIV encephalitis (HIVE) among HIV-infected adults and children is associated with myocardial dysfunction, underscoring the possible connection between cardiac and CNS pathogenesis with HIV-infection (27–29).

Central to CVD and CNS pathologies in PLWH and SIV-infected monkeys is myeloid cell activation. This has been demonstrated in many ways including elevated plasma soluble CD14 (sCD14) and CD163 (sCD163), increased numbers of activated CD14+CD16+ monocytes, and the accumulation of CD163+, CD206+, and MAC387+ macrophages (30–38). Monocyte/macrophage activation and accumulation in the CNS also are correlated with neuroinflammation, encephalitis, and HAND with HIV and SIV infection (26, 32–34, 39–46). Whether such cardiac inflammation and fibrosis, and HAND/HIVE and SIV encephalitis (SIVE) pathogenesis co-occur and correlate with higher levels of monocyte and macrophage activation have not been thoroughly addressed. We and others have shown that blocking monocyte traffic or inhibiting macrophage activation experimentally in SIV-infected macaques reduces cardiac inflammation and CNS pathology, highlighting the importance of monocytes and macrophages in the pathogenesis of both SIV comorbidities (47–49). Plasma galectin-3 and -9, are β-galactoside-binding lectins secreted, inpart, by activated macrophages (50, 51) and correlate with HIV comorbidities (52, 53). Galectin-3 correlates with myocardial fibrosis and cardiac inflammation in HIV-uninfected and HIV-infected cohorts, and plasma and cerebrospinal fluid (CSF) galectin-9 correlates with acute HIV-1 infection and HAND (50, 54–60). Plasma interleukin-18 (IL-18) is an inflammatory cytokine associated with macrophage activation and pyroptosis, plasma viral load, atherosclerosis, and CVD in symptomatic, HIV-infected patients and SIV-infected monkeys (61–64). In addition, sCD163 made solely by myeloid cells correlates with CVD and non-calcified plaque, HAND, and plasma virus in HIV-infected individuals on or off ART and SIV-infected monkeys (30, 31, 39, 65).

In this study, we asked whether SIV infected animals with AIDS codevelop CVD and SIVE, and whether animals that codevelop CVD pathology and SIVE had more monocyte and macrophage activation than animals with CVD pathology alone or SIVE alone, and animals with no significant cardiac pathology and SIV with no encephalitis (SIVnoE). Twenty-three CD8+ T lymphocyte-depleted, SIV-infected macaques with AIDS were examined for the prevalence of cardiac fibrosis, inflammation, and cardiomyocyte degeneration, and SIVE. We assessed the numbers of cardiac macrophages, cardiac collagen, monocyte activation, productive infection in the heart and CNS, and plasma sCD163, IL-18, and galectin-3 and -9. Corollary, translational studies were done in PLWH with and without HAND, where significantly increased numbers of cardiac macrophages were found in HAND versus non HAND individuals.

## Results

### A greater number of animals with AIDS co-develop CVD pathology and SIVE than CVD pathology or SIVE alone

Of twenty-three animals sacrificed with AIDS defining criteria (weight loss, intractable diarrhea, recurrent secondary infections) (**Table 1**), based on histopathology, 10 (43.5%) co-developed CVD pathology (macrophage accumulation, collagen deposition, cardiomyocyte degeneration) and SIVE [SIV-RNA, macrophage accumulation, multi-nucleated giant cells (MNGC)], 6 (26.1%) had CVD pathology or SIVE alone, and 7 (30.4%) had no significant histopathological findings (NSF) and SIV no encephalitis (SIVnoE). Of the sixteen animals with AIDS defining histopathology (**Table 1**), 10 (62.5%) co-developed CVD pathology and SIVE, and 6 (37.5%) had CVD pathology or SIVE alone (**Table 1**). There were greater percentages of animals with a) cardiomyocyte degeneration [NSF and SIVnoE (0/7), CVD-pathology or SIVE alone (1/6) and CVD pathology and SIVE (5/10)]; b) degree of cardiac fibrosis [NSF or SIVnoE (0/7), CVD-pathology or SIVE alone (3/6; 1 severe), CVD pathology and SIVE (6/10; 2 severe)]; and c) cardiac inflammation [NSF and SIVnoE (0/7), CVD pathology or SIVE (3/6, 1 mild) and CVD pathology and SIVE (9/10; moderate-to-severe)] in animals that co-developed CVD pathology and SIVE. There were no significant differences in the average survival days post infection (dpi) among animals with CVD and SIVE (104.8 ± 9.4 dpi), CVD or SIVE alone (99.8 ± 10.9 dpi), and NSF and SIVnoE (168.6 ± 47.2 dpi) nor in age or weight among CVD pathology and SIVE (7.7 ± 0.9 years, 10 ± 0.8 kg), CVD pathology or SIVE alone (6.9 ± 0.7 years, 8.6 ± 1.6 kg), and NSF and SIVnoE (5.7 ± 1.5 years, 7.3 ± 1.2 kg) (**Supplementary Table 1**).

**Table 1:** CVD pathology and SIVE develop together more frequently than does CVD pathology or SIVE alone.

Group	Animal ID	Cardiac inflammation	Cardiac fibrosis	Cardiomyocyte degeneration	CNS pathology
NSF and SIVnoE	186-05	NSF	NSF	NSF	SIVnoE
NSF and SIVnoE	168-05	NSF	NSF	NSF	SIVnoE
NSF and SIVnoE	288-07	NSF	NSF	NSF	SIVnoE
NSF and SIVnoE	FT73	NSF	NSF	NSF	SIVnoE
NSF and SIVnoE	FG73	NSF	NSF	NSF	SIVnoE
NSF and SIVnoE	JR51	NSF	NSF	NSF	SIVnoE
NSF and SIVnoE	JR93	NSF	NSF	NSF	SIVnoE
CVD or SIVE alone	FD37	Mild	Mild	NSF	SIVnoE
CVD or SIVE alone	FC42	Mild	Mild	Moderate	SIVnoE
CVD or SIVE alone	FB92	Mild	Severe	NSF	SIVnoE
CVD or SIVE alone	FD80	NSF	NSF	NSF	SIVE
CVD or SIVE alone	IK28	NSF	NSF	NSF	SIVE
CVD or SIVE alone	KN69	NSF	NSF	NSF	SIVE
CVD and SIVE	JD29	NSF	NSF	Mild	SIVE
CVD and SIVE	JE87	Mild	NSF	NSF	SIVE
CVD and SIVE	DB79	Mild	Mild	NSF	SIVE
CVD and SIVE	FR56	Mild	Mild	Mild	SIVE
CVD and SIVE	LB12	Mild	NSF	Mild	SIVE
CVD and SIVE	KT79	Mild	NSF	NSF	SIVE
CVD and SIVE	CM07	Mild	Moderate	NSF	SIVE
CVD and SIVE	244-96	Moderate	Mild	NSF	SIVE
CVD and SIVE	55-05	Moderate	Severe	Mild	SIVE
CVD and SIVE	FD05	Severe	Severe	Moderate	SIVE

Twenty-three SIV-infected, CD8+ T-lymphocyte depleted rhesus macaques were used in this study, all of which were sacrificed with AIDS. Sections of left ventricular tissue (cardiac tissue) were examined blindly by a veterinary pathologist and the presence and severity of cardiac inflammation, cardiac fibrosis and cardiomyocyte degeneration was determined. Animals were scored with no significant findings (NSF), mild, moderate, or severe CVD pathology. Ten animals were found to have NSF, and 13 animals were found to have CVD pathology. SIVE was diagnosed postmortem and based on the presence of SIV virus in the CNS and MNGC. Thirteen animals had SIVE and ten animals had SIVnoE. Animals were grouped by the presence of CVD and SIVE together (CVD and SIVE, n = 10), one of either CVD or SIVE alone (n = 6), or NSF and SIVnoE (n =7).

### Animals with CVD pathology and SIVE have greater numbers of cardiac macrophages than animals with NSF and SIVnoE

Animals with CVD and SIVE had increased numbers of CD68+ (2.5-fold), CD163+ (2.7-fold), CD206+ (2.5-fold), and MAC387+ (1.8-fold) cardiac macrophages, compared to animals with NSF and SIVnoE (one-way Kruskal-Wallis ANOVA, p<0.05, with Dunn’s multiple comparisons) (**Table 2**). There were similar numbers of cardiac CD163+, CD206+, CD68+, and MAC387+ macrophages in animals with CVD and SIVE compared to animals with CVD or SIVE alone, and similar numbers of cardiac CD3+ T lymphocytes (**Table 2**).

**Table 2:** Animals with CVD pathology and SIVE had increased numbers of cardiac macrophages compared to animals with CVD pathology or SIVE alone, and NSF and SIVnoE animals.

Pathology	CD68+ Macrophage (cells/ mm²)	CD163+ Macrophage (cells/mm²)	CD206+ Macrophage (cells/mm²)	MAC387+ Macrophages (cells/ mm²)	CD3+ T lymphocytes (cells/ mm²
CVD and SIVE	143.1 ± 19.6	325.3 ± 36.8	202 ± 19	31,4 ± 8.8	23.1 ± 4.7
CVD or SIVE alone	107,6 ± 14.6 **	229 ± 34.4 **	116 ± 26.5 **	22.3 ± 5.4 *	22.1 ± 8.3
NSF and SIVnoE	56.6 ± 8.2	122.1 ± 22.9	82.5 ± 16.6	17,4 ± 5.1	10.9 ± 4.2
ANOVA	p< 0.01	p< 0.001	p< 0.01	p< 0.05	p= 0.11

Animals were grouped based on the development of both CVD and SIVE (n=8), CVD or SIVE alone (n=5), or NSF and SIVnoE (n=7). Sections of cardiac tissue were stained immunohistochemically with antibodies recognizing CD163+, CD68+, MAC387+, or CD206+ macrophages (A-D) and CD3+ T-lymphocytes (E). Twenty random, non-overlapping images were sampled at 200x fields of view and the average number of cells/mm^2^ were expressed as plus or minus the SEM. P-values were calculated using a one-way Kruskal-Wallis ANOVA, *p< 0.05, with Dunn’s multiple comparisons (*p<0.05, **p<0.01).

### Animals with CVD pathology and SIVE have greater cardiac collagen deposition than animals with NSF and SIVnoE

**Figure 1:**
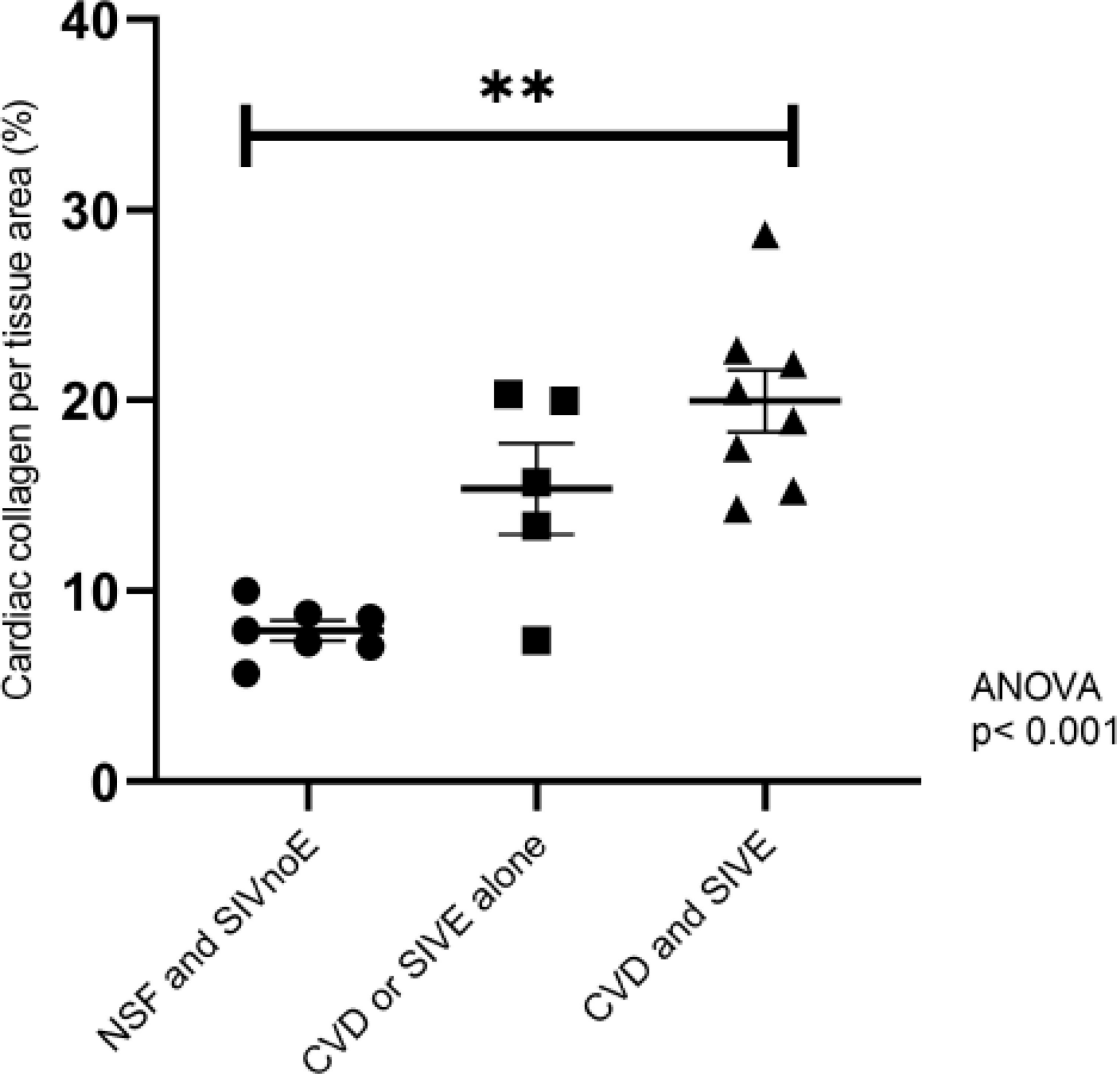
Animals with CVD-pathology and SIVE had a greater percentage of area of cardiac collagen deposition than animals with CVD-pathology or SIVE alone, and NSF and SIVnoE animals. Left ventricle sections from animals grouped as having NSF and SIVnoE (n=7, circles), CVD or SIVE alone (n= 5, squares) and CVD and SIVE (n=8, triangles) were assessed for cardiac collagen deposition using a Massons trichrome stain. Fibrosis was determined as the percentage of collagen per total tissue area and was quantified for each animal using ImageJ Analysis software (non-parametric, one-way ANOVA, Dunn’s multiple comparisons (**p<0.01).

Animals with both CVD and SIVE (2.5-fold; 20 ± 1.5%), and CVD or SIVE alone (1.9-fold; 15.4 ± 2.1%) had similar areas of collagen deposition, but greater percent of collagen deposition than animals with NSF and SIVnoE (7.9 ± 0.5%) (one-way Kruskal-Wallis ANOVA, p<0.05, with Dunn’s multiple comparisons)(**Figure 1**). There were no correlations between the numbers of cardiac macrophages and percent area of cardiac collagen deposition in any of the groups.

### Animals with SIVE alone have greater cardiac inflammation and collagen deposition than animals with SIVnoE

Animals with SIVE alone had greater numbers of CD68+ (1.9-fold), CD163+ (2.1-fold), CD206+ (2.4-fold), and MAC387+ (1.9-fold) cardiac macrophages compared to animals with SIVnoE alone (Mann-Whitney t-test, p<0.05) (**Supplementary Table 2A**). SIVE alone animals had more cardiac collagen deposition (1.8-fold) than SIVnoE animals (Mann-Whitney t-test, p<0.01) (**Supplementary Table 2B**). There were no significant differences between the numbers of cardiac CD3+ T lymphocytes in these groups.

### Animals with both CVD and SIVE have more SIV-RNA+ and SIV-gp41+ cells in the CNS and heart than animals with CVD or SIVE alone, and NSF and SIVnoE animals

**Figure 2:**
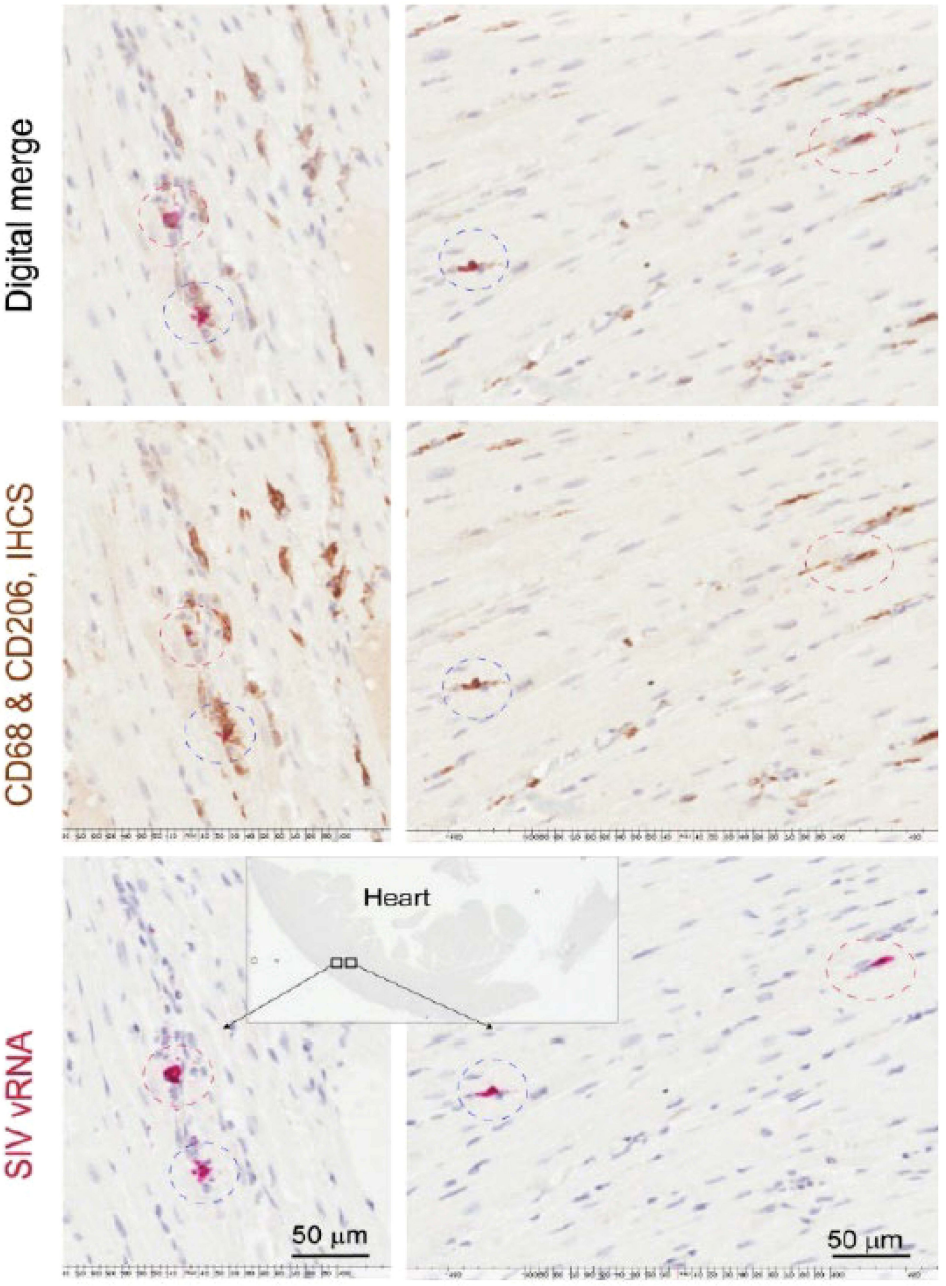
CD68+ and CD206+ cardiac macrophages are SIV-RNA+ in SIV infected monkeys. Sections of left ventricle were stained immunohistochemically for CD68+ and CD206+ macrophages. Cardiac SIV-RNA was detected using RNAscope *in situ* hybridization. Images were digitally merged using Aperio’s Specturm Plus analysis program. Data here are from n=2 animals and representative of n=20 animals.

Overall, we found a greater number of SIV-RNA+ cells (3.8-fold) (Mann-Whitney t-test, p<0.05) and SIV-gp41+ cells (8.2-fold) in the CNS compared to the cardiac tissues in all animals. Animals with CVD pathology and SIVE had a greater number of SIV-RNA+ (3.6-fold) and SIV-gp41+ cells (8.7-fold) in the CNS compared to the heart. Animals with CVD and SIVE had a trend of increased numbers of CNS SIV-RNA+ cells (3.8-fold), CNS SIV-gp41+ cells (1.7-fold), cardiac SIV-RNA+ (2.7-fold), cardiac SIV-gp41+ cells (3.6-fold) than animals with CVD or SIVE alone (**Table 3**). All cardiac SIV-RNA+ cells were CD68+ and CD206+ macrophages not CD3+ T lymphocytes (**Figure 2**).

**Table 3:** Animals with CVD pathology and SIVE had more productively infected cells in the CNS compared to animals with CVD-pathology or SIVE alone, and NSF and SIVnoE animals.

Pathology	Cardiac SIV-DNA+ (cells/mm²)	CNS SIV-RNA+ (cells/mm²)	Cardiac SIV-RNA+ (cells/mm²)	CNS SIV-gp41+ (cells/mm2)	Cardiac SIV-gp41+ (cells/mm²)
CVD and SIVE	0.4 ± 0.2	19.3 ± 2.3] *	5.40 ± 2.4	31.5 ± 11.2	3.6 ± 1.9
CVD or SIVE alone	0.7 ± 0.4	5.1 ± 3.1	0.45 ± 0.4	18.5 ± 8.8	0
NSF and SIVnoE	1.7 ± 0.3	0.7 ± 0.2	2.7 ± 1.9	8.1 ± 2.3	4.1 ± 1.3
ANOVA	p= 0.24	p< 0.05	p= 0.20	p= 0.67	p= 0.15

Animals were grouped based on the development of both CVD and SIVE, CVD or SIVE alone, and NSF and SIVnoE. The average number of SIV-DNA+ cells in the heart and, SIV-RNA+ and SIV-gp41+ cells in CNS cortical and cardiac tissues are reported. One section of center ventricle and three sections of CNS cortical from SIV-infected macaques (n=10) were assessed for SIV-RNA+ cells/mm2. Measurements of CNS SIV-RNA+ cells/mm2 were determined by averaging counts from three CNS cortical sections. One section of CNS cortical and cardiac tissues (n=13) were assessed for SIV-gp41+ cells/mm2 plus or minus SEM. Twenty, random, non-overlapping 400x fields of view were sampled for each section per animal and the average number of SIV-gp41+ cells/mm2 were determined. P-values were calculated using a one-way Kruskal-Wallis ANOVA, *p< 0.05, with Dunn’s multiple comparisons. (*p<0.05).

### Animals with SIVE alone have increased numbers of CNS SIV-RNA+ macrophages compared to SIVnoE animals

Animals with SIVE alone had increased numbers of CNS SIV-RNA+ cells (18.4-fold), but similar, low numbers of cardiac SIV-RNA+ cells and SIV-gp41+ cells, and CNS SIV-gp41+ cells compared to animals with SIVnoE alone (Mann-Whitney t-test p<0.01) (**Supplementary Table 3**). Animals with CVD-pathology alone had similar numbers of CNS SIV-RNA+ cells, cardiac SIV-RNA+ cells, and CNS SIV-gp41+ cells compared to NSF animals (**Supplementary Table 3**).

### Animals with CVD-pathology and SIVE have greater numbers of CD14+CD16+ monocytes compared to NSF and SIVnoE animals

**Figure 3:**
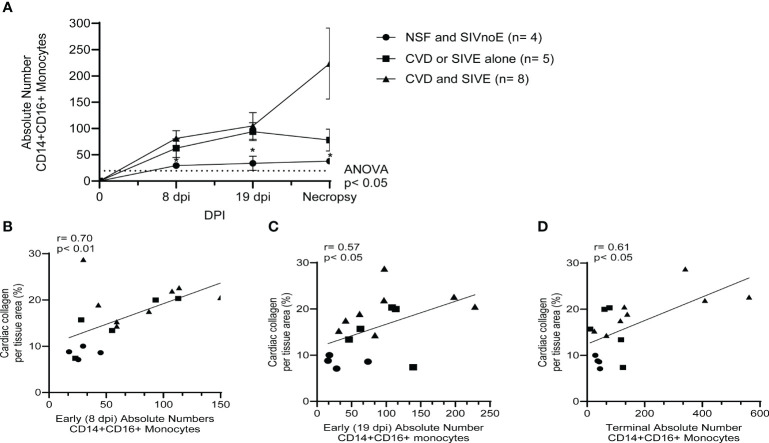
Animals with CVD and SIVE had increased numbers of CD14+CD16+ monocytes early and terminally compared to animals with CVD or SIVE alone, and NSF and SIVnoE animals. **(A)**. Absolute numbers of CD14+CD16+ monocytes, as determine by flow cytometry and CBC, were assessed early (8 dpi and 19 dpi) and terminally in animals with NSF and SIVnoE (n=4, circle), CVD or SIVE alone (n= 5, square and CVD and SIVE (n=8, triangle). The pre-infection baseline was 19.38 CD14+CD16+ monocytes (one-way ANOVA, *post-hoc*, non-parametric, Mann-Whitney t-test, *p<0.05 **(B-D)**. The absolute numbers of CD14+CD16+ monocytes at early infection (8 dpi and 19 dpi) and terminally from animals with NSF and SIVnoE (n=4, circle), CVD or SIVE alone (n= 5, square), and CVD and SIVE (n= 8, triangle) positively correlated with the percent cardiac collagen per tissue area (non-parametric, Spearman’s correlation, p< 0.05).

Animals with CVD and SIVE had increased numbers of CD14+CD16+ monocytes early [8 dpi (81.3 ± 13.4 CD14+CD16+ monocytes; 2.8-fold) and 19 dpi (104.8 ± 23.6 CD14+CD16+ monocytes; 3.1-fold)] and terminally (223.6 ± 63.1 CD14+CD16+ monocytes; 5.9-fold) compared to animals with NSF and SIVnoE (29.5 ± 5.1, 34 ± 11.7, and 37.8 ± 3.1 CD14+CD16+ monocytes, respectively) (one-way Kruskal-Wallis ANOVA, *p<0.05, with Dunn’s multiple comparisons) (**Figure 3A**). Animals with CVD and SIVE had similar numbers of CD14+CD16+ monocytes early (8 dpi; 1.3-fold) and a trend of increased CD14+CD16+ monocytes terminally (2.9-fold) compared to CVD-pathology or SIVE alone animals (62.5 ± 15.9 and 78.1 ± 18.6 respectively) (**Figure 3A**). There was a correlation between the numbers of CD14+CD16+ monocytes early (8 dpi; r= 0.70, p<0.01 and 19 dpi; r= 0.57, p<0.05) and terminally (r= 0.61, p<0.05) with cardiac collagen deposition (Spearman’s correlation, p<0.05) (**Figures 3B–D**).

### Animals with CVD and SIVE have increased plasma biomarkers associated with monocyte and macrophage activation

**Figure 4:**
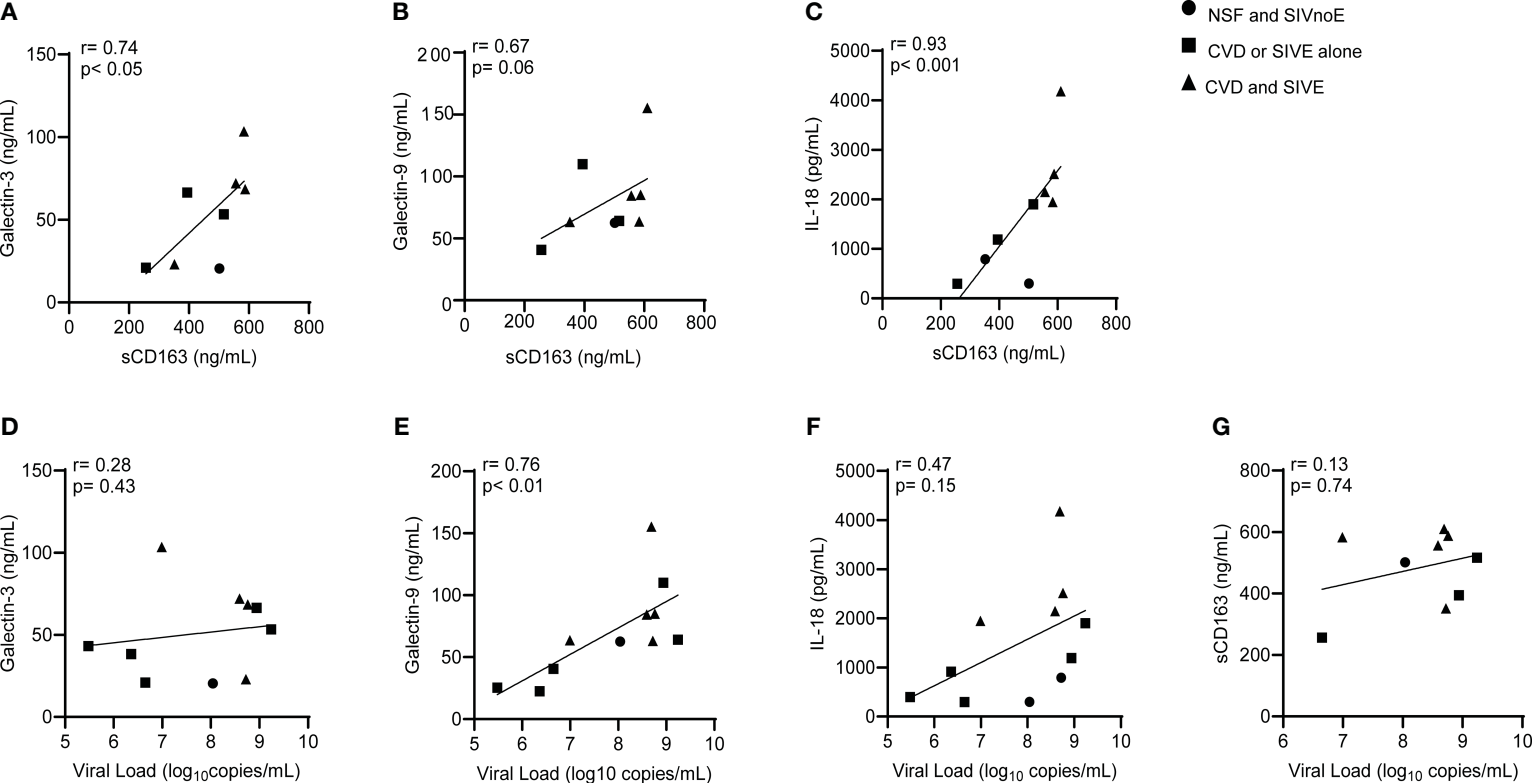
Plasma sCD163 correlates with galectins- 3 and -9, and IL-18 in SIV infected monkeys. **(A-C)**. Spearman’s correlation was used to assess the relationship between terminal levels of plasma galectins -3 and -9, and IL-18, and terminal plasma sCD163. **(D-G)**. Spearman’s correlation was used to assess the relationship between terminal levels of plasma galectins-3 and -9, IL-18, and sCD163, and plasma viral load. Data generated here were from n=23 animals. P-values accepted at Spearman’s p< 0.05.

Animals with CVD and SIVE had a trend of increased plasma sCD163 (2.6-fold) and IL-18 (2.4-fold), galectin-3 (1.2-fold) and galectin-9 (1.5 fold) compared to animals with CVD or SIVE along, and NSF and SIVnoE animals (one-way Kruskal-Wallis ANOVA, *p<0.05, with Dunn’s multiple comparisons) (**Table 4**). Plasma sCD163 positively correlated with galectin-3 (r= 0.74, p<0.05) and IL-18 (r= 0.93, p<0.001), and trended to correlate with galectin-9 (r= 0.67, p= 0.06). Consistent with previous studies in HIV infected individuals (60, 66), there was a positive correlation between plasma galectin-9 and plasma viral load (r= 0.76, p<0.01) (Spearman’s correlation, p<0.05) (**Figure 4**) but there were no significant correlations between plasma virus and galectin-3, IL-18, and sCD163. Animals with CVD-pathology alone had a trend of increased plasma sCD163 (2.2-fold) and similar levels of IL-18, galectin-3, and galectin-9 compared to NSF animals (Mann-Whitney t-test p<0.05) (**Table 5**). SIVE alone animals had greater plasma IL-18 (2.9-fold) and a trend of increased galectin-3 (1.5-fold) and galectin-9 (2.1-fold). There were similar levels of sCD163 between animals with SIVE and SIVnoE (Table 5). Animals with CVD-pathology alone had similar levels of sCD163, IL-18 and galectin-9 compared as NSF animals (**Table 5**).

**Table 4:** Plasma sCD163, IL-18, galectin -3 and -9 are higher in animals with CVD-pathology and SIVE than animals with CVD–pathology or SIVE alone.

Pathology	Plasma sCD163 (ng/mL)	Plasma IL-18 (pg/mL)	Plasma galectin-3 (ng/mL)	Plasma galectin-9 (ng/mL)
**CVD and SIVE**	879 ± 232	1859 ± 500	60.2 ± 11.4	76.5 ± 14.7
**CVD or SIVE alone**	334 ± 76.7	1018 ± 250	50.3 ± 8.6	52.5 ± 16.1
**NSF and SIVnoE**	1052 ± 551	299	20.5	62.6
**ANOVA**	p= 0.07	p= 0.27	p= 0.24	p= 0.6

Terminal plasma sCD163, IL-18, galectin-3 and galectin-9 were measured in animals with NSF and SIVnoE, CVD or SIVE alone, and CVD and SIVE. ELISAs were performed according to the manufacturer’s protocol. P-values were calculated using a one-way Kruskal-Wallis ANOVA, *p< 0.05, with Dunn’s multiple comparisons (*p<0.05).

**Table 5:** Animals with SIVE alone had more plasma sCD163, IL-18, and galectin-3 and -9 than animals with SIVnoE.

SIVE Pathology	Plasma sCD163 (ng/mL)	Plasma IL-18 (pg/mL)	Plasma galectin-3 (ng/mL)	Plasma galectin-9 (ng/mL)
**SIVE**	782 ± 200	1814 ± 385] *	55.9 ± 9.7	75 ± 12.9
**SIVnoE**	689 ± 309	619 ± 183	37.8 ± 8.5	37.8 ± 9.2
Cardiac Pathology	Plasma sCD163 (ng/mL)	Plasma IL-18 (pg/mL)	Plasma galectin-3 (ng/mL)	Plasma galectin-9 (ng/mL)
**CVD**	779 ± 200	1437 ± 362	48.9 ± 8.3	66.7 ± 12.1
**NSF**	697 ± 312	1202 ± 473	51.2 ± 17.2	63.4 ± 0.8

Terminal plasma sCD163, IL-18, galectin-3 and galectin-9 were measured in animals with SIVnoE or SIVE alone., and NSF or CVD alone. P-values were calculated using non-parametric Mann-Whitney T-tests with significance accepted at p< 0.05. NSF, no significant findings. CVD-pathology, cardiovascular pathology. SIVnoE, SIV with no encephalitis. SIVE, SIVE encephalitis (*p<0.05).

### HIV-infected individuals with HIVE have greater cardiac inflammation and fibrosis than HIVnoE individuals

**Figure 5:**
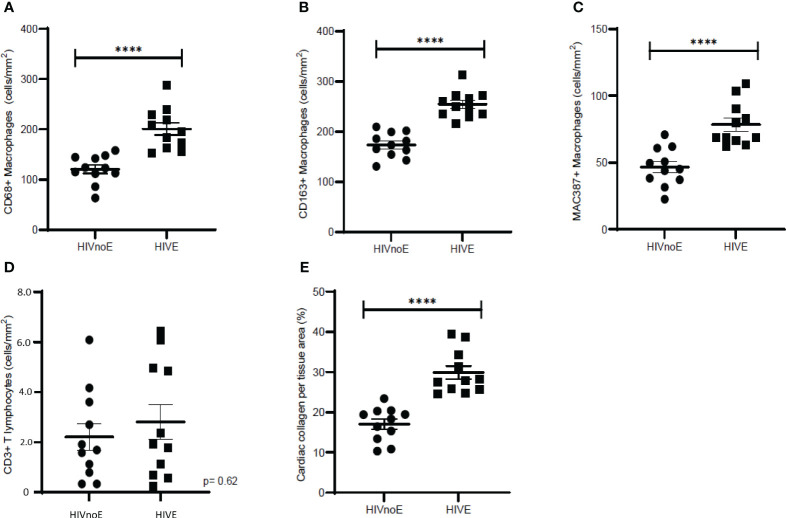
Individuals with HIVE had greater numbers of cardiac macrophages and collagen deposition than HIVnoE individuals. **(A-D)**. Increased CD68+, CD163+, and MAC387+ cardiac macrophages in individuals with HIVE. Trend of increased numbers of cardiac CD3+ T lymphocytes regardless of HIVE. Sections of cardiac tissue were stained immunohistochemically with antibodies recognizing CD68+, CD163+, or MAC387+ macrophages, and CD3+ T-lymphocytes. Twenty random, non-overlapping images were sampled at 200x fields of view, and the data was expressed as the average number of cells/mm^2^ plus or minus the SEM. **(E)**. Increased cardiac collagen deposition in individuals with HIVE. Cardiac collagen deposition was assessed using a Masson’s trichrome stain. Fibrosis was determined as the percentage of cardiac collagen per total tissue area and was quantified for using ImageJ Analysis software with twenty random, non-overlapping images sampled at 200x fields of view. Cardiac tissues were supplied by the Manhattan HIV Brain Bank. Data was presented as the average percentage of collagen plus or minus the SEM. P-values were calculated using a nonparametric, Mann-Whitney t test, HIVnoE (circles); HIV with no encephalitis. HIVE (squares); HIV encephalitis (^****^p<0.0001).

We next sought to determine whether HIV-infected individuals with HIVE (n =11) had increased cardiac inflammation and fibrosis over that seen in HIV infected individuals without encephalitis (HIVnoE) (n =11) (**Table 6**). In age-, race- and sex-matched individuals, we found that the HIVE group had greater numbers of CD68+ (1.7-fold, 200.62 ± 12.41 cells), CD163+ (1.5-fold, 254.29 ± 8.05 cells), and MAC387+ (1.7-fold, 78.52 ± 4.94 cells) cardiac macrophages compared to individuals with HIVnoE (120.49 ± 8.44 CD68+ macrophage, 173.42 ± 7.55 CD163+ macrophage, and 46.58 ± 4.29 MAC387+ macrophage) (Mann-Whitney t test, p<0.05) (**Figures 5A-C**). There were similar numbers of cardiac CD3+ T lymphocytes in individuals with HIVE (28.15 ± 6.97 cells) compared to individuals with HIVnoE (22.11 ± 5.39 cells) (**Figure 5D**). Additionally, there was a higher percentage of cardiac collagen deposition (1.8-fold, 29.87 ± 1.63%) in the HIVE group compared to the HIVnoE group (17.06 ± 1.26%) (Mann-Whitney t test, p<0.05) (**Figure 5E**). These data are similar to the results we find in SIV infected monkeys, supporting the translational nature of the monkey data.

**Table 6:** Patients from the Manhattan HIV Brain Bank (MHBB) were examined for the prevalence of HIVE.

MHBB ID	AGE	SEX	RACE	CD4+ T lymphocytes (< 200 cells/mL)	HIVE
MHBB552	47	m	W	YES	HIVnoE
MHBB558	51	m	h	YES	HIVnoE
MHBB625	48	m	b	YES	HIVnoE
010003	45	m	W	NO	HIVnoE
010011	42	m	h	YES	HIVnoE
MHBB532	47	m	h	YES	HIVnoE
010171	45	m	b	YES	HIVnoE
MHBB533	41	m	h	NO	HIVnoE
030025	46	m	b	YES	HIVnoE
020025	37	m	h	YES	HIVnoE
030024	43	m	b	YES	HIVnoE
MHBB509	46	m	W	NO	HIVE
MHBB519	50	m	h	YES	HIVE
MHBB540	47	m	b	YES	HIVE
010017	43	m	W	YES	HIVE
010026	37	m	h	YES	HIVE
010065	46	m	h	YES	HIVE
010070	37	m	b	YES	HIVE
010103	40	m	h	YES	HIVE
010129	43	m	b	YES	HIVE
010231	47	m	h	YES	HIVE
030015	43	m	b	YES	HIVE

Twenty-two HIV infected males from the MHBB were assessed. Eleven individuals had HIV with no encephalitis (HIVnoE) and an average age of 44.7± 1.15 years. Nine HIVnoE individuals had a CD4+ T lymphocyte count <200 cells. Eleven individuals have HIV encephalitis (HIVE) and an average age of 43.5± 1.27 years. Ten HIVE individuals had a CD4+ T lymphocyte count <200 cells. Each individual with HIVE was matched in sex, race, and age to an individual with HIVnoE.

## Conclusion- Discussion

Monocyte and macrophage activation and accumulation in the heart or the CNS are consistently correlated with the development of CVD and CVD pathology, HAND and HIVE, and SIVE (22, 67–70). Less is known or reported about the frequency that CVD, CVD pathology, HIVE and HAND with AIDS in humans and animal models, and in PLWH (27–29), or whether co-development is associated with increased monocyte activation, macrophage accumulation, or HIV and SIV infection. Here, we find that animals with AIDS co-developed CVD pathology and SIVE more frequently than CVD pathology or SIVE alone, and individuals with HIVE have increased numbers of cardiac macrophages and fibrosis compared to age- and sex-matched non-HIVE controls. We report that animals that co-developed CVD-pathology and SIVE have higher numbers of CD14+CD16+ monocytes, and plasma sCD163, IL-18, and galectin-3 and -9, and cardiac macrophage accumulation/collagen deposition than animals with CVD or SIVE alone, and NSF and SIVnoE animals. These observations support the notion of increased monocyte activation and cardiac macrophage accumulation with the co-development of cardiac inflammation and fibrosis and SIVE, and underscore the translational findings in the monkey studies with those in HIV infected individuals.

Macrophages are key regulators of fibrogenesis through their interactions with myofibroblasts, wound healing responses, and production of profibrotic factors like galectin-3, osteopontin, and transforming growth factor- beta (TGF-β) (71–74). We and others have previously shown that CD163+ and CD206+ cardiac macrophage accumulation and monocyte activation correlates with cardiac inflammation and fibrosis with HIV and SIV infection (38, 75–77). Similarly, CD163+ and CD206+ perivascular macrophages and 5-bromo-2’-deoxyuridine-labeled (BrdU+) MAC387+ macrophage accumulation in the CNS are major components of HIVE and SIVE lesions (43–46). These observations are consistent with the notion that macrophage accumulation in the heart and CNS correlate with cardiac and SIVE pathogenesis. Our findings extend those observations to suggest that higher levels of monocyte activation, biomarkers of myeloid cell activation in plasma, and macrophage accumulation in the heart correlate with the co-development of cardiac inflammation and fibrosis and SIVE. We have previously shown, by blocking macrophage accumulation with the anti-alpha-4 integrin antibody (47, 48), the polyamine biosynthesis inhibitor methylglyoxal-bis-guanylhydrazone (MGBG) (37) or minocycline (49) correlates with decreased cardiac and CNS inflammation, cardiac fibrosis and tissue histopathology further supporting the role that macrophage accumulation has in the development of CVD alone and SIVE alone (37). Here, we report that animals with CVD and SIVE had more CD68+, CD163+, CD206+, and MAC387+ cardiac macrophages, and cardiac collagen deposition than animals with CVD or SIVE alone, demonstrating that concomitant CVD-pathology and SIVE correlates with higher levels of cardiac macrophage activation, plasma markers of myeloid activation and cardiac fibrosis than CVD-pathology or SIVE alone. We report that animals with CVD alone had more cardiac macrophages and collagen deposition than animals with NSF alone, consistent with previous reports (71, 76, 78). We demonstrate parallel observations in an HIVE cohort compared to age- and sex-matched controls with HIV infection without HIVE, that have increased cardiac macrophages and collagen. Increased cardiac collagen deposition is linked to cardiac macrophage accumulation, although we did not find a statistically significant correlation between macrophage accumulation and percent collagen in the CVD-pathology and SIVE groups. It is possible that we did not find a correlation between the numbers of cardiac macrophages and cardiac collagen in this study due in part to the CD8+ T lymphocyte-depletion model of rapid AIDS. SIV infected macaques with CD8+ T lymphocyte-depletion are more likely to develop AIDS and SIVE and CVD-pathology within 3-4 months, as opposed to 1-3 years, but do not develop chronic cardiovascular diseases (79, 80). Indeed, Shannon et al. (2000) found that acutely infected rhesus macaques did not develop contractile dysfunction and cardiac pathology when compared to chronically infected macaques (81), suggesting that rapid AIDS pathogenesis in macaques does not consistently result in severe cardiomyopathy. We found that animals with SIVE alone had a greater number of cardiac macrophages and collagen deposition than animals with SIVnoE alone, and we found higher numbers of cardiac macrophages and fibrosis in individuals with HIVE, than age and sex matched HIV infected controls without HIVE. Overall, these findings suggest that increased cardiac macrophage accumulation and fibrosis correlate with HIVE in HIV infected individuals and SIVE in SIV infected macaques. Kuroda et al. (2019), demonstrated that CD163+ and CD206+ cardiac macrophages are the most abundant cardiac macrophage subsets in uninfected rhesus macaques with severe cardiac inflammation (82). We report that CD163+ and CD206+ cardiac macrophage subsets are the most abundant cardiac in SIV infected macaques with AIDS. We found that animals with CVD-pathology and SIVE have higher numbers of CD163+ and CD206+ cardiac macrophages than animals with NSF and SIVnoE, indicating that CD163+ and CD206+ cardiac macrophage subsets are correlated with the severity of CVD and SIVE pathologies. Similarly, we find that CD163+ cardiac macrophages are the most abundant cardiac macrophage subset in HIV infected individuals with HIVE, suggesting that CD163+ cardiac macrophage accumulation is associated with HIVE pathogenesis.

We found that higher numbers of activated CD14+CD16+ monocytes occur with the co-development of CVD pathology and SIVE. The CD14+CD16+ monocyte subset normally comprises 5-10% of the total monocyte population, but their expansion with SIV infection and AIDS correlates with the development of CVD-pathology alone, or SIVE alone (32, 69, 83–85). Moreover, animals with CVD and SIVE had greater numbers and percentages of CD14+CD16+ monocytes early in infection and terminally compared to CVD or SIVE alone animals, and animals with NSF and SIVnoE, suggesting that CD14+CD16+ monocyte activation is a biomarker of AIDS pathogenesis and concomitant CVD pathology and SIVE similar to CVD with HIV and HAND in humans (67, 86–88). Prior reports have shown that CD14+CD16+ monocytes are increased/associated with HAND alone (89), and CVD-pathology alone (35, 90). These blood monocytes are thought to be a mature subset of activated monocytes (84, 91) that are persistently activated and are more susceptible to HIV and SIV infection (92, 93). Indeed, early infection and trafficking of C-C chemokine receptor 2 (CCR2)-positive CD14+CD16+ monocytes into the CNS correlates with the development of HIVE and SIVE (93–95). Further, increased CD14+CD16+ monocyte activation correlates with cardiovascular and cerebrovascular inflammation in HIV infected individuals on ART, suggesting that monocyte activation persists despite ART and is linked to the development of CVD and vasculopathy with infection (36, 90, 96). Here, we show that numbers of CD14+CD16+ monocytes early and terminally also correlate with the percentage of cardiac collagen deposition in all animals with AIDS, indicating that increased CD14+CD16+ monocytes correlate with cardiac fibrogenesis. Together our findings suggest that the development of concomitant CVD-pathology and SIVE with AIDS is correlated with increased levels of CD14+CD16+ monocyte activation, and plasma biomarkers of monocyte activation.

We find that animals with concomitant CVD-pathology and SIVE had more SIV-RNA+ and SIV-gp41+ cells in the CNS and heart than animals with CVD-pathology or SIVE alone, and NSF and SIVnoE animals. We note that in all SIV infected monkeys with AIDS, there are far fewer SIV-RNA+ and SIV-gp41+ cells in the heart than the CNS suggesting that macrophage accumulation more so than SIV-RNA+ and SIV-gp41+ cells, are linked to the co-development of CVD pathology and SIVE. This remains the case when plasma virus is undetectable with ART because both CVD-pathology, and HAND persists in the post-ART era, and correlates with markers of monocyte/macrophage activation like plasma sCD163 and sCD14, IL-18, galectin -3 and -9 (30, 39, 41, 97). This is consistent with previous studies showing few SIV-RNA+ cells in the heart regardless of the severity of cardiac inflammation (76, 98). Conversely, other studies have shown that myocardial SIV-RNA correlates with diastolic dysfunction (99, 100). We found that cardiac SIV-RNA+ cells are CD68+CD206+ macrophages and not CD3+ T lymphocytes in all animals, suggesting that a small population of macrophage are productively infected in the heart. We found little to no SIV-DNA+ latently infected cells in the heart. We postulate that the difference of magnitudes in SIV-DNA+, SIV-RNA+, and SIV-gp41+ cells is likely due to the sensitivity of the assays used. We and others have previously shown that productively infected, CD14+CD163+ perivascular macrophages and multinucleated giant cells (MNGCs) comprise the main population of infected macrophages in the brain and correlate with the development of SIVE lesions (43, 46, 69, 83, 101, 102). Overall, our findings support the notion that animals with concomitant CVD-pathology and SIVE have more SIV-RNA+ macrophages and SIV-gp41+ productively infected cells in the heart and CNS than animals with CVD-pathology or SIVE alone.

We found higher levels of biomarkers of plasma sCD163 and IL-18 in animals with CVD and SIVE compared to animals with CVD or SIVE alone, and animals with NSF and SIVnoE consistent with the notion that concomitant CVD and SIVE is correlated with higher monocyte/macrophage activation, and numbers of CD14+CD16+ monocytes. We and others have previously reported that increased plasma sCD163 correlates with non-calcified coronary plaque (30), HAND (39), and all-cause mortality (65) in HIV infected individuals on ART, and in SIV infected rhesus macaques (39, 103). Similarly, plasma IL-18 is produced by macrophages and is also increased with atherosclerosis and CVD in HIV infected individuals (64, 104, 105), and SIV infected macaques (63). Increased NLR Family Pyrin Domain-Containing 3 (NLRP3) inflammasome activation occurs in macrophages with HIV-infection and drives IL-18 and IL-1β production. NLRP3 inflammasome activation is correlated with macrophage activation and pyroptosis (106, 107), disease progression in gut-associated lymphoid tissues (62), neuroinflammation (108, 109) and atherosclerosis in HIV infected individuals (110). This data, and that of others show higher levels of plasma IL-18 in animals that co-developed CVD-pathology and SIVE, suggesting that NLRP3 inflammasome activation may drive both cardiac inflammation and SIVE pathogenesis. We also found similar levels of plasma galectin-3 and -9 in all animals regardless of pathology. Other studies have shown that plasma galectin-3 is increased with HIV-infection (54) and correlates with non-calcified coronary plaque in HIV infected individuals (55); and increased plasma galectin-9 correlates with acute HIV-infection (59, 60, 66), neurocognitive dysfunction (58), and all-cause mortality (111). We found increased galectin-3 and -9 in animals with SIVE alone animals compared to SIVnoE animals. Studies in mice show that increased galectin-3 expression in the brain correlates with microglia activation and neuroinflammation post-CNS injury (112–115), suggesting that there may be a connection between increased galectin-3 and SIVE pathogenesis. We did not find increased plasma galectin-3 in animals with CVD alone likely because of the acute nature of our rapid AIDS model. Previous studies have shown that increased plasma galectin-3 correlates with cardiac inflammation and fibrosis in the uninfected population (116–121) and may correlate with cardiac pathogenesis in HIV infected individuals (54, 55, 68). Our findings indicate that plasma galectin-3 and -9, biomarkers of myeloid cell activation, are higher in animals with SIVE and may correlate with the severity of AIDS pathologies. We also find that plasma sCD163 correlates with plasma galectin-3 and -9, and IL-18 in all animals, and plasma galectin-9, but not sCD163, galectin-3, and IL-18, correlates with plasma viral load. This is consistent with recent reports showing that plasma galectin-9 correlates with plasma viral load in HIV infected individuals (66). Together, these data demonstrate that plasma IL-18 and galectin-3 and -9, are better correlated with monocyte/macrophage activation than plasma viral load. Our findings are consistent with previous data from the Multicenter AIDS Cohort Study (MACS) showing that subclinical atherosclerosis and cognitive dysfunction are correlated with increased biomarkers of monocyte/macrophage activation despite plasma HIV suppression with ART (88, 122, 123).

The concept of the “heart-brain axis,” in which pathogenesis in the heart and CNS are linked, has been discussed in the uninfected population (17, 20, 124), but has not been thoroughly studied with HIV- or SIV- infection. In this study, we report that animals with AIDS co-developed CVD and SIVE and had higher levels of CD14+CD16+ monocyte activation, plasma biomarkers of myeloid cell activation, cardiac inflammation and fibrosis, and SIV-RNA+ and SIV-gp41+ cells in the CNS and heart than animals with CVD or SIVE alone, and animals with NSF and SIVnoE. We also show that cardiac SIV-RNA+ cells are CD68+CD206+ cardiac macrophage. Importantly, we show that HIV infected individuals with HAND also have more cardiac inflammation and fibrosis than individuals with no HAND. This study sheds further light on the importance of monocyte and macrophage activation in AIDS pathogenesis, and suggests that the development of future therapies in HIV infected individuals should target and inhibit myeloid cell activation in the heart and CNS together.

## Materials and methods

### Animals, SIV-infection, and CD8+ T-lymphocyte depletion

Twenty-three rhesus macaques were utilized in this study. Five were housed at Harvard University’s New England Primate Research Center (NEPRC) and eighteen were housed at Tulane University’s Tulane National Primate Research Center (TNPRC) in accordance with standards of the American Association for Accreditation of Laboratory Animal Care. This was a retrospective study using all male Rhesus macaques (Macaca mulatta). Animals were experimentally infected intravenously (i.v) by inoculation with bolus of SIVmac251 viral swarm (20 ng of SIV p28) provided by Ronald Desrosiers, over a 5 minute time-period. Animals were adult (3.6-12.6 years old). Animals with the Mamu B^*^08 and B^*^17 alleles were excluded. Blood samples were taken prior to, on the day of infection, and weekly thereafter. Animals underwent CD8+ T-lymphocyte depletion for rapid AIDS and consistent SIVE. CD8+ T-lymphocyte depletion was achieved with subcutaneous administration of human anti-CD8 antibody, cM-T807 (10 mg/kg) at day 6 post-infection, and i.v. administration (5 mg/kg) on days 8 and 12 post-infection. Simian AIDS was determined postmortem by the presence of opportunistic infections, tumors, and the development of SIV giant cell pneumonia, cytomegalovirus pneumonia, SIVE with giant cells, *pneumocystis jirovecii*, or lymphoma. With the presence of AIDS, animals were anesthetized with ketamine-HCl and euthanized with i.v. pentobarbital overdose and exsanguinated.

### Plasma viral load

Plasma SIV-RNA was quantified using real-time PCR, as previously describe (125–127). Five hundred µL of EDTA plasma was collected and SIV virions were pelleted by centrifugation at 20,000 g for 1 hour. The PCR assay targets conserved sequences of SIV-*gag* The threshold sensitivity was 100 copy Eq/mL, with an average inter-assay coefficient variation of less than 25%. The CT cut off for SIV DNA is 39-40 cycles.

### Assessment of inflammation and fibrosis in cardiac tissues and CNS SIVE

Following exsanguination, a standard necropsy was performed and lymph nodes and parenchymal organs including heart and brain, were fixed in 10% neutral buffered formalin. Tissues were paraffin embedded, sectioned at 5µm, and stained with hematoxylin and eosin. Sections of cardiac (left ventricle) and central nervous system (CNS) cortical tissues were analyzed blindly by a veterinary pathologist. Ten randomly selected images of cardiac and CNS cortical tissues were taken using an Olympus BX43 Light Microscopy (Evident, Tokyo, Japan) at 400x fields and graded subjectively and blindly by an ACVP certified Veterinary Pathologist, and categorized based on the degree of cardiac inflammation, cardiac fibrosis, and cardiomyocyte degeneration as having: A) no significant findings (NSF), B) mild, C) moderate, or D) severe pathology. SIVE was diagnosed based on the presence of MNGCs, accumulation of perivascular macrophages, and productive SIV infection (103, 128–130).

### Single-label immunohistochemistry of cardiac tissues

Sections of formalin-fixed, paraffin-embedded cardiac tissues were immunohistochemically assessed for numbers of macrophages and CD3+ T-lymphocytes, as previously described (48). Macrophages were identified using monoclonal antibodies against CD163 (clone EdHu-1, Serotec; Oxford, UK), CD68 (clone KP1, Dako; Glostrup, Denmark), Myeloid/Histiocyte Antigen (clone MAC387, Dako), and CD206 (clone 685645, R&D Systems; Minneapolis, MN); T-lymphocytes were identified using a polyclonal antibody against CD3 (Agilent- cat A0452, Dako, Santa Clara, CA). Data are presented as the mean positive number of cells/mm^2^ from 20 non-overlapping fields of view plus or minus the standard error of the mean (SEM). SIV-productively infected cells in cardiac and CNS cortical tissues were immunohistochemically stained for SIV-gp41+ cells (clone: KK41, NIH AIDS Reagent Program; Germantown, MD). Quantitation of SIV-gp41+ cells/mm^2^ was achieved by counting SIV-gp41+ cells from 20 random, non-overlapping images of brain and cardiac sections at 400x total magnification using the Zeiss Axio Imager.M1 microscope and AxioVision (Version 4.8, Zeiss; Oberkochen, Germany).

### Measurement of myocardial fibrosis

Cardiac collagen deposition was measured using a modified Massons Trichrome stain, as previously described (48). Tissue sections were imaged using a Zeiss Axio Imager M1 microscope with Plan-Apochromat x20/0.8 Korr objectives. The percent collagen per total tissue area was determined using ImageJ Analysis software from 20 non-overlapping 200x microscopic fields (field area= 0.148mm^2^) and data are presented as the percent collagen per total tissue area plus or minus the SEM.

### Immunohistochemical analysis of samples from the Manhattan HIV Brain Bank cohort

Sections of cardiac tissues were examined post-mortem, from n=22 individuals from the Manhattan HIV Brain Bank (MHBB) cohort. All HIV infected individuals were ART naïve and matched with regard to age, race, and sex. All individuals examined were male. Eleven patients had HIV no encephalitis (HIVnoE) and eleven patients had HIV encephalitis (HIVE). HIVnoE patients had an average age of 44.7 ± 1.15 years, and HIVE patients had an average age of 43.5 ± 1.27 years. Four patients were white, ten were Hispanic, and eight were black. Nine HIVnoE patients, and ten HIVE patients had CD4+ T cell counts < 200 cells. Single-label immunohistochemistry was performed on formalin-fixed, paraffin-embedded sections of cardiac tissues. Macrophages were identified with monoclonal antibodies against CD68 (KP1, Bio-RaD), CD163 (EDHU, Bio-Rad), CD206 (MMR/CD206, RD Systems), and Myeloid/Histiocyte Antigen MAC387 (MAC387, Bio-Rad) cardiac macrophages. Cardiac T-lymphocytes were identified using the polyclonal antibody against CD3. Data are presented as the mean number of positive cells/mm^2^ from 20 non-overlapping 200x fields of view plus or minus the SEM. Cardiac collagen deposition was measured using a modified Massons Trichrome stain. Tissue sections were imaged using a Zeiss Axio Imager M1 microscope with Plan-Apochromat x20/0.8 Korr objectives. The percent collagen per total tissue area was determined using ImageJ Analysis software from 20 non-overlapping 200x microscopic fields (field area = 0.148mm^2^) and data are presented as the percent collagen per total tissue area plus or minus the SEM.

### SIV-RNA and SIV-DNA detection using RNAscope and DNAscope

SIV-RNA was detected *in situ* using the RNAscope ^®^ 2.5 HD Assay-Red (Advanced Cell Diagnostics [ACD]; Newark, CA) on formalin-fixed, paraffin-embedded, 5-µm thick sections of three CNS cortical and one cardiac tissue per animal, as previously described (131). Sections were deparaffinized in xylenes and 100% ethanol and air dried. Antigen retrieval with boiling citrate buffer for 15 min and protease digestion at 40°C for 30 min was performed (131). SIV-RNA-specific probes targeting SIVmac239 envelope (ACD), RNAscope^®^ positive control Mmu-PPIB (ACD) or RNAscope^®^ negative control DapB (ACD) were applied to sections. Quantitation of SIV-RNA+ cells/mm^2^ in the CNS and heart were achieved by counting and averaging the numbers of SIV-RNA+ cells from 20 random, non-overlapping images taken from three CNS cortical sections and one cardiac section, respectively, at 400x total magnification using the Zeiss Axio Imager.M1 microscope and AxioVision. SIV-DNA was detected *in situ* using an SIV-DNA sense probe (ACD) for RNAscope^®^ Assay on one cardiac section per animal. DNAscope was performed, as previously described (25, 132). To reduce non-specific signal, heart tissues were pre-treated with 2N HCL for 30 min at room temperature. Following DNAscope, cardiac sections were scanned and digitized with Aperio CS2 Scanscope. SIV-DNA+ cells/mm^2^ were quantified using a positive pixel count algorithm in Aperio’s Spectrum Plus analysis program (version 9.1, Aperio ePathology Solutions, Leica Biosystems; Wetzlar, Germany), as previously described (133).

### Plasma biomarker ELISAs

Plasma IL-18 (R & D Systems; Minneapolis, MN), galectin-3 (R & D Systems), galectin-9 (R & D Systems) and soluble CD163 (sCD163) (IQ Products; Groningen, Netherlands) concentrations were analyzed with ELISAs according to the manufacturer’s protocol. Concentrations of galectin-3 and galectin-9 were measured using BioTek Powerwave 340 (BioTek; Winooski, VT) at a wavelength of 450 nm and a correction wavelength of 540 nm. Concentrations of plasma galectin-3 and -9, and sCD163 were presented as ng/mL. Concentrations of plasma IL-18 were presented as pg/mL.

### Flow cytometry

Flow cytometric analysis was conducted on 100 μl aliquots of whole blood collected in EDTA-coated tubes. Blood samples were taken at 0, 8, 19 days post infection (dpi) and terminally. Samples from animals housed at the NERPC were shipped and analyzed the same day and samples from animals at the TNPRC were shipped overnight. Erythrocyte lysis was performed (ImmunoPrep Reagent System, Beckman Coulter; Brea, CA), followed by 2 washes with PBS, and incubation with fluorochrome-conjugated antibodies including anti-CD14-APC (clone: M5E2, BD Pharmingen; San Diego, CA), anti-CD16-PE (clone: 3G8, BD Pharmingen) anti-HLA-DR-PerCP-Cy5.5 (clone: L243, BD Pharmingen). All samples were fixed in 2% paraformaldehyde and results were acquired on a BD FACS Aria (BD Biosciences; San Jose, CA) and analyzed with Tree Star Flow Jo version 8.7. Monocytes were first selected based on size and granularity (FSC vs SSC) followed by selection of HLA-DR^+^ CD14^+^ cells. From this acquisition gate the percentage of monocyte subsets expressing CD14 and/or CD16 could be determined. The absolute number of peripheral blood monocytes for each animal was calculated by multiplying the total white blood cell count by the total percentage of monocytes determined by flow cytometry.

### Statistical analysis

Statistical analyses were done using Prism version 10 software (GraphPad Software, Inc.; San Diego, CA). Comparisons between animals with CVD and SIVE, animals with CVD or SIVE alone, and NSF and SIVnoE animals were made using a one-way analysis of variance (ANOVA) with Dunn’s multiple comparisons. Comparisons between CVD only and NSF only, and SIVE only and SIVnoE only animals were made using a non-parametric Mann-Whitney *t*-test. A Spearman rank test was used for all correlations. Statistical significance was accepted at p< 0.05.

### Study approval

Animals were housed at Harvard University’s New England Regional Primate Research Center (NEPRC) or Tulane University’s National Primate Center (TNPRC) and handled in strict accordance with Harvard University’s and Tulane University’s National Primate Research Center Institutional Animal Care and Use Committee (IACUC). Animal IACUC approval from NEPRC and TNPRC was granted for all procedures: The NERPC protocol number for this study was 04420 and the animal welfare assurance number was A3431-01. The TNRPC the protocol number is 3497 and the animal welfare assurance number is A4499-01. All human cardiac tissues from the Manhattan HIV Brain Bank cohort were obtained and examined with the written informed consent of all individuals prior to participation in this study. These were de-identified post mortem tissue specimens and were therefor IRB exempt.

## Data availability statement

The raw data supporting the conclusions of this article will be made available by the authors, without undue reservation.

## Ethics statement

The studies involving humans were approved by Manhattan HIV Brain Research IRB. The studies were conducted in accordance with the local legislation and institutional requirements. The human samples used in this study were acquired from another research group. Written informed consent for participation was not required from the participants or the participants’ legal guardians/next of kin in accordance with the national legislation and institutional requirements. The animal study was approved by Harvard Universities New England Primate Research Center and Tulane Universities National Primate Research Center. The study was conducted in accordance with the local legislation and institutional requirements.

## Author contributions

KSW Wrote manuscript; conducted immunohistochemical detection of cardiac and CNS SIV-gp41+ cells; performed *in situ* detection of cardiac and CNS SIV-RNA+ cells, measured levels of plasma IL-18, sCD163, galectins-3 and -9; and performed correlations for plasma cytokines. JAW wrote manuscript, conducted immunohistochemical analysis of cardiac macrophages, lymphocytes, cand cardiac collagen deposition; performed correlations between cardiac collagen deposition and monocytes. JW Conducted immunohistochemical analysis of cardiac macrophages, lymphocytes, cand cardiac collagen deposition; performed correlations between cardiac collagen deposition and monocytes. PA performed flow cytometric analysis of CD14+CD16+ monocytes. AM graded the prevalence and severity of gross cardiac pathology and SIVE. RB, QL, and W-KK conducted *in situ* detection of cardiac SIV-DNA+ cells; determined co-localization of cardiac SIV-RNA+ cells and CD68+/CD206+ cardiac macrophages. W-KK wrote and edited manuscript. KCW principal investivator conceived of study, analyzed data and wrote manuscript. All authors contributed to the article and approved the submitted version.

## Supplementary material

Supplementary Table 1 Characteristics of the rhesus macaques examined in this study. N=23 SIV-infected, CD8+ T-lymphocyte depleted rhesus macaques were used in this study, all of which were sacrificed with AIDS. Sections of left ventricular tissue (cardiac tissue) were examined blindly by a veterinary pathologist and the presence and severity of cardiac histopathology was scored as no significant cardiac pathology (findings) (NSF) or cardiac pathology was present (CVD). SIV encephalitis (SIVE) was diagnosed postmortem and based on the presence of SIV virus in the CNS and MNGC. All animals were males and were euthanized following the development of simian AIDS.

Supplementary Table 2 Animals with SIVE alone had greater numbers of cardiac macrophages and cardiac collagen deposition compared to animals with SIVnoE. Animals were grouped based on SIVnoE alone (n=10) and SIVE alone (n=10). **(A)**. Sections of cardiac tissue from all animals were stained immunohistochemically with antibodies recognizing CD163+, CD68+, MAC387+, or CD206+ macrophages and CD3+ T-lymphocytes. Twenty random, non-overlapping images were sampled at 200x fields and the average number of cells/mm^2^ were calculated and expressed as plus or minus the SEM. **(B)**. Left ventricle sections were assessed for cardiac collagen deposition using a Massons trichrome stain. The percentage of collagen per total tissue area was averaged from 20 non-overlapping 200x fields of view and expressed as the average plus or minus the standard error of the mean. Fibrosis, determined as the percentage of collagen per total tissue area, was quantified for each animal using ImageJ Analysis software. P-values were calculated using a non-parametric, Mann-Whitney t-tests with significance accepted at p<0.05.

Supplementary Table 3 Animals with SIVE alone had greater numbers of SIV-RNA+ and SIV-gp41+ cells in the CNS. Animals were grouped based on the development of CVD and no significant findings (NSF) in cardiac tissues, or SIVE alone and SIVnoE. The average number of cardiac SIV-DNA+ cells, SIV-RNA+ and SIV-gp41+ cells in CNS cortical and cardiac tissues were reported plus or minus the SEM. One section of left ventricle was assessed for cardiac SIV-DNA+ cells in animals with CVD (n=4) compared to NSF (n=3), and in animals with SIVE (n=5) compared to SIVnoE (n=2). One section of left ventricle was assessed for cardiac SIV-RNA+ cells in animals with CVD (n=6) compared to NSF (n=3), and in animals with SIVE (n=5) compared to SIVnoE (n= 4). Three sections of CNS cortical tissues were assessed for CNS SIV-RNA+ cells in animals with SIVE (n=7) compared to SIVnoE (n=4), and in animals with CVD (n=8) compared to NSF animals (n=3). One section of left ventricle was assessed for cardiac SIV-gp41+ cells and were compared between animals with CVD (n=8) and animals with NSF (n=4), and between animals with SIVE (n=7) and animals with SIVnoE (n=5). One section of CNS cortical tissue was assessed for CNS SIV-gp41+ cells and were compared in animals with SIVE (n= 8) versus SIVnoE (n =5), and in animals with CVD (n=9) versus NSF (n=4). P-values were calculated using a nonparametric, Mann-Whitney t-test, p< 0.05, (*p<0.05, **p<0.01, ***p<0.001, ****p<0.0001).
