# Supplementary material for: Simian immunodeficiency virus-infected rhesus macaques with AIDS co-develop cardiovascular pathology and encephalitis
Source: Front Immunol. 2023 Oct 27;14:1240946. doi: 10.3389/fimmu.2023.1240946 (PMC10641955; doi:10.3389/fimmu.2023.1240946)
Supplement: Supplementary Table 1 — Characteristics of the rhesus macaques examined in this study. N=23 SIV-infected, CD8+ T-lymphocyte depleted rhesus macaques were used in this study, all of which were sacrificed with AIDS. Sections of left ventricular tissue (cardiac tissue) were examined blindly by a veterinary pathologist and the presence and severity of cardiac histopathology was scored as no significant cardiac pathology (findings) (NSF) or cardiac pathology was present (CVD). SIV encephalitis (SIVE) was diagnosed postmortem and based on the presence of SIV virus in the CNS and MNGC. All animals were males and were euthanized following the development of simian AIDS. [file Table_1.docx]

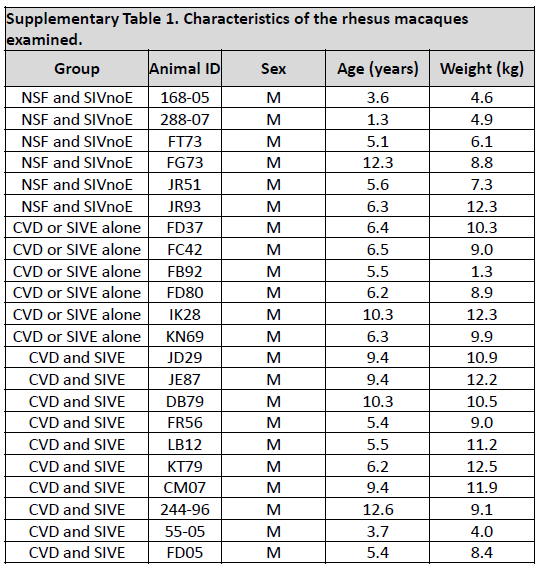
**Supplementary Tables**

**Supplementary Table 1. Characteristics of the rhesus macaques examined in this study.** N=23 SIV-infected, CD8+ T-lymphocyte depleted rhesus macaques were used in this study, all of which were sacrificed with AIDS. Sections of left ventricular tissue (cardiac tissue) were examined blindly by a veterinary pathologist and the presence and severity of cardiac histopathology was scored as no significant cardiac pathology (findings) (NSF) or cardiac pathology was present (CVD). SIV encephalitis (SIVE) was diagnosed postmortem and based on the presence of SIV virus in the CNS and MNGC. All animals were males and were euthanized following the development of simian AIDS.


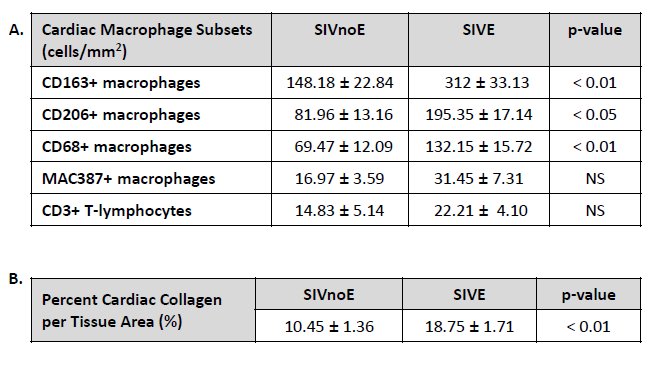


**Supplementary Table 2. Animals with SIVE alone had greater numbers of cardiac macrophages and cardiac collagen deposition compared to animals with SIVnoE.** Animals were grouped based on SIVnoE alone (n=10) and SIVE alone (n=10). (A). Sections of cardiac tissue from all animals were stained immunohistochemically with antibodies recognizing CD163+, CD68+, MAC387+, or CD206+ macrophages and CD3+ T-lymphocytes. Twenty random, non-overlapping images were sampled at 200x fields and the average number of cells/mm^2^ were calculated and expressed as plus or minus the SEM. (B). Left ventricle sections were assessed for cardiac collagen deposition using a Massons trichrome stain. The percentage of collagen per total tissue area was averaged from 20 non-overlapping 200x fields of view and expressed as the average plus or minus the standard error of the mean. Fibrosis, determined as the percentage of collagen per total tissue area, was quantified for each animal using ImageJ Analysis software. P-values were calculated using a non-parametric, Mann-Whitney t-tests with significance accepted at p<0.05.

**
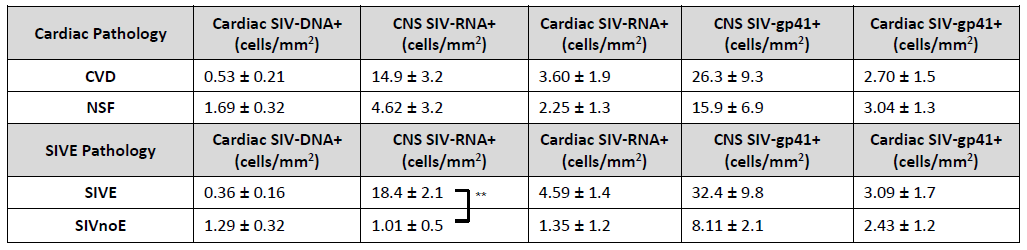
**

**Supplementary Table 3. Animals with SIVE alone had greater numbers of SIV-RNA+ and SIV-gp41+ cells in the CNS.** Animals were grouped based on the development of CVD and no significant findings (NSF) in cardiac tissues, or SIVE alone and SIVnoE. The average number of cardiac SIV-DNA+ cells, SIV-RNA+ and SIV-gp41+ cells in CNS cortical and cardiac tissues were reported plus or minus the SEM. One section of left ventricle was assessed for cardiac SIV-DNA+ cells in animals with CVD (n=4) compared to NSF (n=3), and in animals with SIVE (n=5) compared to SIVnoE (n=2). One section of left ventricle was assessed for cardiac SIV-RNA+ cells in animals with CVD (n=6) compared to NSF (n=3), and in animals with SIVE (n=5) compared to SIVnoE (n= 4). Three sections of CNS cortical tissues were assessed for CNS SIV-RNA+ cells in animals with SIVE (n=7) compared to SIVnoE (n=4), and in animals with CVD (n=8) compared to NSF animals (n=3). One section of left ventricle was assessed for cardiac SIV-gp41+ cells and were compared between animals with CVD (n=8) and animals with NSF (n=4), and between animals with SIVE (n=7) and animals with SIVnoE (n=5). One section of CNS cortical tissue was assessed for CNS SIV-gp41+ cells and were compared in animals with SIVE (n= 8) versus SIV (n=5), and in animals with CVD (n=9) versus NSF (n=4). P-values were calculated using a nonparametric, Mann-Whitney t-test, p< 0.05, (*p<0.05, **p<0.01, ***p<0.001, ^****^p<0.0001).
